# The chemopreventive effects of native Brazilian plants on stomach cancer: A review of the last 25 years

**DOI:** 10.18632/oncoscience.618

**Published:** 2025-05-08

**Authors:** Iara Lopes Lemos, Maria Josiane Macedo, Ana Paula da Fonseca Machado, Roberto de Paula do Nascimento, Lívia Mateus Reguengo, Valeria Helena Alves Cagnon, Mario Roberto Marostica Junior

**Affiliations:** ^1^Laboratory of Nutrition and Metabolism, School of Food Engineering, University of Campinas, Campinas 13083-862, São Paulo, Brazil; ^2^Department of Structural and Functional Biology, Institute of Biology, University of Campinas (UNICAMP) Campinas 13083-862, São Paulo, Brazil

**Keywords:** bioactive compounds, gastric cancer, phenolic compounds, cytotoxic

## Abstract

Stomach cancer (SC) is the fifth most prevalent and deathly type of cancer worldwide. This is a multifactorial disease, and its development can be influenced by both genetic factors and dietary habits. On the other hand, a regular consumption of fruit and vegetables rich in bioactive compounds, such as polyphenols and flavonoids, has demonstrated anti-inflammatory, antioxidant, and chemopreventive effects on SC. Brazil, which has a vast plant diversity, appears to be a promising scenario for investigating species with potential anti-tumor action. Thus, the objective of this review is to present and discuss the chemopreventive aspects of native Brazilian species in SC. Less-explored fractions of native plants, such as açaí (*Euterpe oleracea*), araçá-do-campo (*Psidium guineense*), yellow araçá (*Psidium cattleianum Sabine*), cacao (*Theobroma cacao*), coriander (*Eryngium foetidum*), physalis (*Physalis angulata*), guava (*Psidium guajava*), jambu (*Acmella oleracea*), pitanga (*Eugenia uniflora*), and ubaia (*Eugenia patrisii*), have demonstrated the ability to slow down the progression of the disease, indicating suppression of cell proliferation and survival, induction of apoptosis, and regulation of the cell cycle, despite showing not mechanism of action in the great majority of these studies. Although, still little studied, Brazilian plant matrices could show a promising impact against SC.

## INTRODUCTION

Global data for 2022 shows a worrying scenario, with approximately 20 million new cases of cancer are diagnosed, with approximately 10 million deaths attributed to the disease [1]. It is estimated that the annual incidence of cancer could reach 35 million cases by 2050, reflecting an increase of 77% in relation to the rates recorded in 2022 [1]. Specifically, in relation to stomach cancer (SC) or gastric cancer, more than 968,000 new diagnoses were reported in 2022, with around 660,000 deaths, consolidating this neoplasm as the fifth most incident and lethal in the world. SC is a common malignancy, representing a public health problem worldwide [1]. In Brazil, according to the National Cancer Institute (INCA), between 2023 and 2025 there will be approximately 704,000 new cases of cancer [2]. Specifically, for SC, the projection is for 21,480 new diagnoses, with a higher incidence among men (13,340 cases) compared to women (8,140 cases). With regard to mortality, data for 2020 points to 13,850 deaths from SC in the country, which is equivalent to a rate of 6.54 deaths per 100,000 inhabitants. The impact of the disease is more pronounced among men, with 8,772 deaths (8.47 per 100,000), while among women there were 5,078 deaths, corresponding to a rate of 4.69 per 100,000 inhabitants [2].

**Figure 1:**
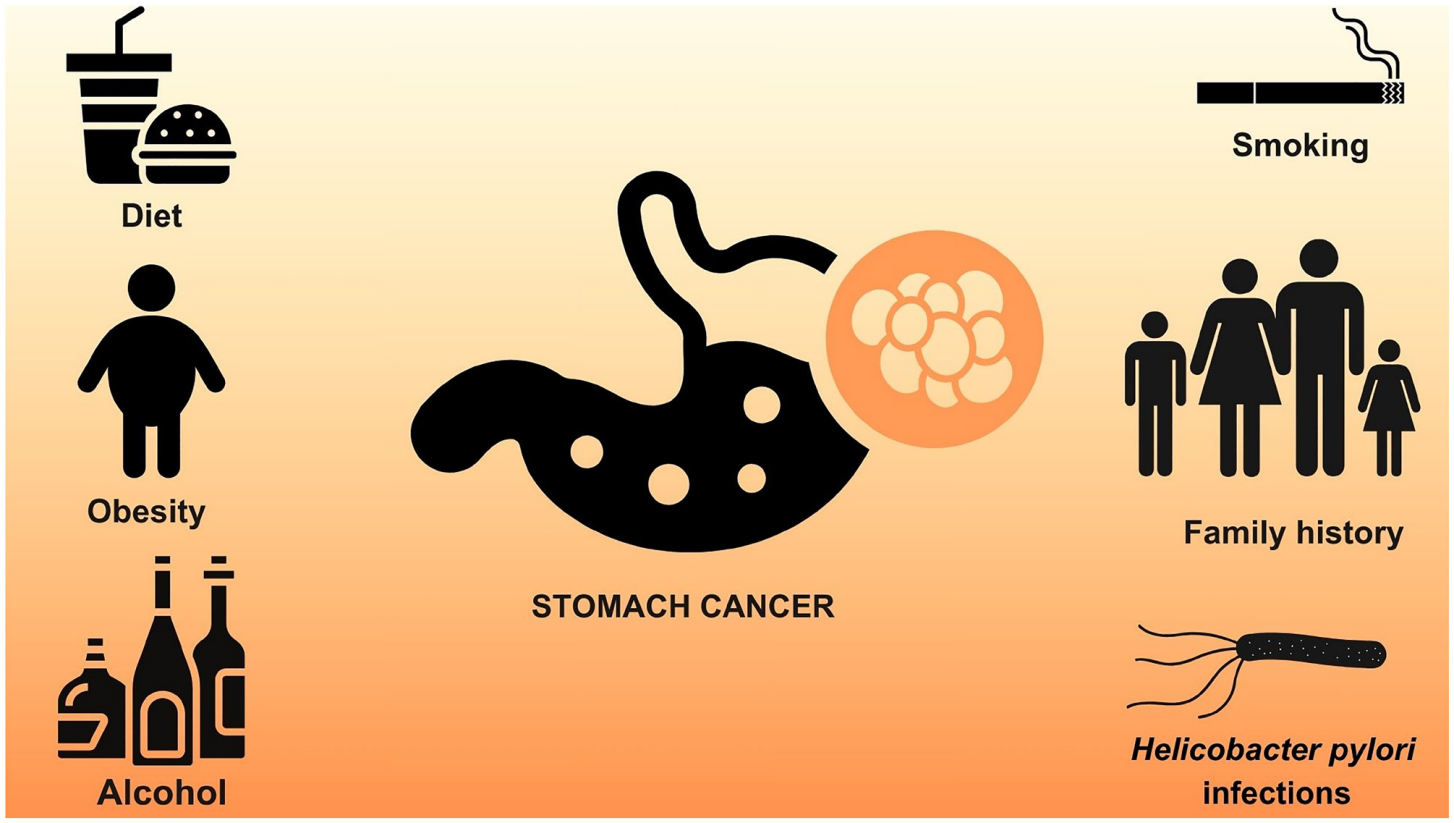
Risk factors for developing stomach cancer. Some factors can contribute to the development of stomach cancer, such as alcohol consumption, smoking, obesity, diet (high consumption of sodium and smoked products), family history and *H. pylori* infections. Own authorship, using illustrations from canva pro.

SC can be classified as papillary, tubular, mucinous, or poorly cohesive carcinoma [3]. According to Lauren’s histological classification, which is considered the most popular, SC can be divided into intestinal, diffuse, or mixed [4]. That said, SC is considered a multifactorial disease, in which several factors can influence its development, such as family history, alcohol consumption, smoking, *Helicobacter pylori* infections, and poor eating habits (Figure 1) [5, 6]. High consumption of sodium and smoked foods is known to increase the risk of developing SC, while the consumption of fruits and vegetables has a protective effect against the disease. Plant matrices contain various phytochemical compounds with anti-inflammatory, antioxidant, and anticancer activities, which explains their protective effect [7].

In the early stages of SC, there are usually no symptoms, but in the advanced stages, manifestations such as abdominal pain, anorexia, weight loss, and persistent vomiting are common [8]. Thus, early detection is fundamental in order to reduce the mortality rate, since high levels of deaths from SC are associated with late diagnosis, due to the absence of symptoms. It is known that the therapeutic method in SC must be chosen for each patient according to the stage of the disease [9]. In the early stage of SC, tumor resection is the most recommended therapy and, in the advanced stage, chemotherapy [10]. SC therapies such as tumor resection, chemotherapy, immunotherapy, and radiotherapy are invasive and promote undesirable effects in patients such as nausea, vomiting, and gastrointestinal disorders [11, 12]. Therefore, investing in alternative therapies is an interesting choice for reducing these symptoms. In this sense, products obtained from natural sources have shown positive effects in the prevention and treatment of cancer [13]. These chemopreventive effects found in some plant matrices are mainly correlated to the presence of secondary metabolites such as phenolic compounds [14].

Brazil is a country with an extensive diversity of fruits and plant species, which consequently also includes a variety of bioactive compounds. Polyphenols are phytochemicals found in native Brazilian flora, in addition to protecting the plants, these compounds also have antioxidant, anti-inflammatory, and anti-cancer properties in humans [15]. Some plant matrices from other regions of the world, such as China, Japan and India, have already demonstrated antiproliferative and apoptosis-inducing effects in SC, such as *Morus Alba* leaves on a SNU-601 cell line, *Scutellaria baicalensis* extract induced apoptosis in BGC-823 and MGC-803, and *Tripterygium wilfordii* exerted cytotoxic effects on gastric carcinoma xenografts [16]. An extract obtained from the leaves, fruits, and roots of *Asimina triloba* demonstrated significant antitumor effects on AGS cells, promoting the inhibition of cell growth, the induction of apoptosis, and the interruption of the cell cycle in the Sub-G1 phase [17]. Despite Brazil’s rich and valuable biodiversity, many native plant species remain little explored by the scientific community [18]. Studies exploring Brazilian plant matrices associated with SC are limited and often require an in-depth mechanistic approach. However, these initial investigations offer a promising basis for more detailed and enlightening future research [15]. Thus, the aim of this review is to present and discuss the chemopreventive aspects of native Brazilian species in SC.

## APPLICATION OF NATIVE BRAZILIAN SPECIES IN STOMACH CANCER

**Figure 2:**
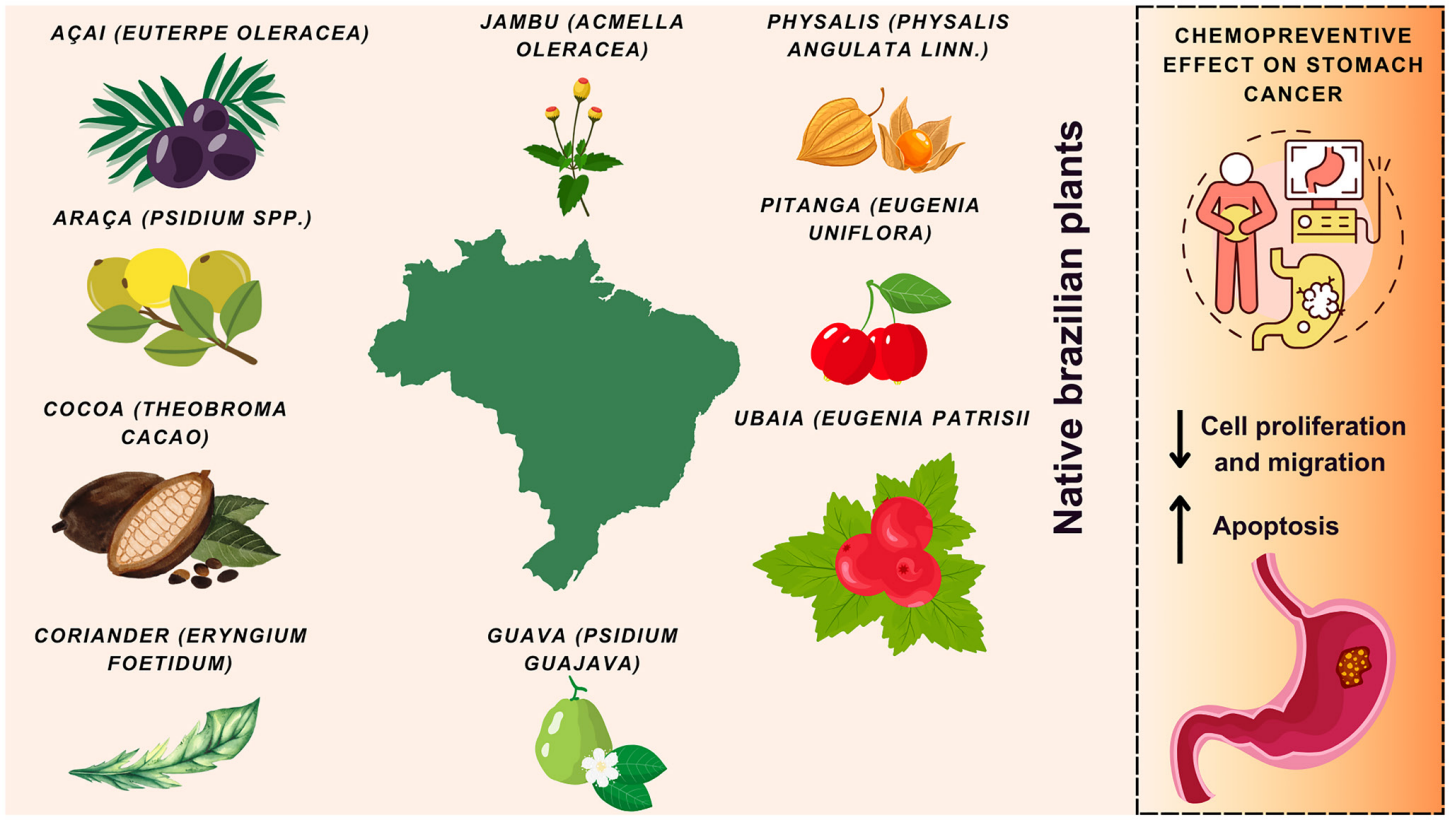
Brazilian plants exhibit chemopreventive effects in stomach cancer, reducing cell proliferation and migration and increasing apoptosis. Own authorship, using illustrations from canva pro.

The species were selected based on a combination of criteria, such as their native occurrence in Brazil, presence of bioactive compounds already described in the literature and preliminary data related to stomach cancer. The official list of native Brazilian plants, published in 2018 by the Ministry of the Environment (Native Species of Brazilian Sociobiodiversity of Food Value, 2018), was used to guide the choice of species. The search was carried out in databases of high scientific rigor, including Scopus, SciELO, PubMed and ScienceDirect. In all, 14 articles were selected and analyzed. The species investigated include açaí (*Euterpe oleracea*), araçá-do-campo (*Psidium guineense*), yellow araçá (*Psidium cattleianum Sabine*), cacao (*Theobroma cacao*), coriander (*Eryngium foetidum*), physalis (*Physalis angulata*), guava (*Psidium guajava*), jambu (*Acmella oleracea*), pitanga (*Eugenia uniflora*), and ubaia (*Eugenia patrisii*). Among the plant fractions, the leaves were the most studied. Cell culture studies were the most frequently used, with only two studies conducted on an animal model and no clinical studies were carried out. Figure 2 summarizes the findings reported in the literature about Brazilian species and their relationship with SC. Table 1 summarizes the results from the studies with Brazilian plants on SC. In the following topics, we discuss these findings in depth, contextualizing their impacts and implications for SC.

**Table 1:** Effects of native Brazilian plants on stomach cancer based on experimental studies

Products	Scientific name(S)	Composition	Experimental model	Dosage and period	Results	References
Clarified açaí pulp extract	*Euterpe oleracea* Mart	Orientin: 38 mg Homoorientin: 25 mg, Taxifolin deoxyhexose: 31 mg; Cyanidin 3-glucoside: 18 mg, Cyanidin 3-rutinoside: 45 mg.	AGP01 cell line	Resazurin reduction assay for 24 and 72 h: 6.25 μg/mL, 12.5 μg/mL, 25 μg/mL, 50 μg/mL, 100 μg/mL and 200 μg/mL. Comet assay, cell death and wound healing assay for 72 h: 50 μg/mL, 100 μg/mL and 200 μg/mL.	↓: cell viability (20,73%.) No genotoxic damage. ↓: cell migration No necrosis or apoptosis was induced.	[21]
Freeze-dried açaí pulp	*Euterpe oleracea* Mart	–	C57BL/6 mice	Diet supplemented with 5% and 10% açaí for 4, 24 and 52 weeks.	Açaí did not inhibit gastric carcinogenesis	[24]
Araçá leaves; chloroform fraction of extracts with 80% methanol.	*Psidium cattleianum* sabine	Identified compounds: Polymethoxylated flavone, sesquiterpenes, genistein, ferulic acid, oleanolic acid and 3’, 4’, 5’ trimethoxyflavone.	SNU-16 cell line	25, 50, 100 e 200 μg/mL for 72 h.	↓: cell viability ↑: cells in the sub G1 phase ↓: Bcl-2 ↑: Caspase-8 and caspase-3	[33]
Araçá leaves; β-caryophyllene oxide isolated.	*Psidium cattleianum* sabine	Chemical composition analyzes were not performed	SNU-1 and SNU-16 cells line	25, 50, 125, 625, 3125 μM for 4 days	Cytotoxic activity: IC50: 16.79 μM for SNU-1 and IC50: 27.39 μM for SNU-16	[44]
Araçá leaves from different regions of Pará, Brazil, essential oils (Pgui-1 and Pgui-2).	*Psidium guineense*	Identified compounds: Monoterpene hydrocarbons: Pgui-1: 58.1% and Pgui-2: 54.3% Limonene monoterpenes: Pgui-1: 30.2% and Pgui-2: 30.4% α-pinene: Pgui-1: 22.5% and Pgui-2: 17.7% E-caryophyllene: Pgui-1: 2.1% and Pgui-2: 0.1%.	AGP01 cell line	0.4 and 25 μg/mL for 72h.	Antioxidant activity: Pgui-1: 11.5% and Pgui-2: 27.7% inhibiting of the DPPH. Cytotoxic activity: Pgui-1: IC50: 8.2 μg/mL. Pgui-2: IC50:15.7 μg/mL.	[40]
Cocoa bean husk, 50% ethanol extract (v/v) and 50% acetone extract (v/v)	*Theobroma cacao*	TPC: 13,3%-14,0%	SNU1 cell line	100, 200, 400, and 800 μg/mL for 48 h.	Proliferation inhibition from 400 μg/mL dose	[48]
Coriander, ethanolic extract	*Eryngium foetidum*	No compounds identified	SGC-7901, MGC-803, AGS and GES-1 cells line	10, 25, 50, 100, 150, 200, 250, 300 μg/mL for 72 h.	cytotoxic effect on SGC-7901 cells ↑: cells in the sub G1 phase ↓: cells in the S and G2/M phases. ↓: cyclin D1, CDK4. ↑: p21 ↑:Cyt C, Bax, cleaved caspase-3. ↓:Bcl-2	[54]
Guava leaves, essential oil	*Psidium guajava*	Identified compounds: Sesquiterpenic hydrocarbons (42.6%), Oxygenated sesquiterpenoids (44.7%), Epi-β-bisabolol sesquiterpenes (16.1%), ar-curcumene (9.8%), β), β-bisabolene (9.2%), E caryophyllene (5.1%) and caryophyllene oxide (4.5%).	AGP01 cell line	0.4 and 25 μg/mL for 72h.	Antioxidant activity: 38,6% inhibiting of the DPPH. Cytotoxic effect IC50: 16.3 μg/mL.	[40]
Jambu, hydroethanolic extract	*Acmella oleracea (L.)*	Spilantol isolated by HPLC	AGP01 cell line	1,25, 2,5, 5,0, 10,0, 20,0, 40,0 and 80,0 μg/mL for 24, 48 and 72 h.	Proliferation inhibition IC50: 21.84 μg/mL	[61]
Jambu, isolated compound spilanthol	*Acmella oleracea (L.)*	–	AGP01 cell line	1,25, 2,5, 5,0, 10,0, 20,0, 40,0 and 80,0 μg/mL for 24, 48 ans 72 h.	Proliferation inhibition IC50: 11,86 μg/mL Molecular docking: interact favorably with JAK1 and JAK2	[62]
Physalin B isolated from physalis	*Physalis angulata L*	–	SGC-7901 and HGC-27 cells line	5, 10, 20, 30 and 40 μM/mL for 24, 48 and 72 h.	Inhibited cell viability in HGC-27 cells. IC50: 9 μM/mL for 72 h. Induced cell cycle arrest in the G0/G1 phase. ↓: cyclin D1, cyclin D3, CDK4, CDK6 and cyclin E. **↑:** cleaved caspase-8, caspase-3, caspase-7 and PARP	[65]
Physapubescin B isolated from physalis	*Physalis angulata L.*	–	Six-week-old nude mice.	40 mg per kg of body weight	Reduction in tumor volume: 31,4 %	[66]
Withangulatin I, isolated from the methanolic extract of Physalis	*Physalis angulata L.*	–	AGS cell line		↓: cell viability IC50: 1,8 μM.	[67]
Pitanga leaves; essential oil	*Eugenia uniflora*	Identified compounds: Oxygenated sesquiterpenes: 20.8–69.0%, Sesquiterpene hydrocarbons: 18.0 53.9%, Curzerene: 13.4–50.6%, Selina-1,3,7(11)-trien-2-one: 18.1–43.1%, Germacrene B: 5.0–18.4%, Caryophyllene oxide: 1.2–18.1%, (E)-caryophyllene: 0.3–9.1%, β-elemene: 3.5–8.9%, γ-elemene: 2.0 7.8%	AGP01 cell line	0.4–25 μg /mL for 72 h.	Antioxidant activity: 30.3–45.1% inhibiting of the DPPH. Cytotoxic activity: IC50: 15.42 μg/mL	[71]
Pitanga leaves; ethanolic extract, ethyl acetate fraction and aqueous fraction.	*Eugenia uniflora*	Quinic Acid, Myricitrin, and Myricetin-3- O -(2”- O -galloyl)-α-L-rhamnopyranoside, ellagitannins and Oenotein B.	AGS cell line	3.125 - 800 μg/mL for 48 h.	Only the aqueous extract reduces cell viability. IC50: 178 μg/mL.	[72].
Ubaia leaves and roots, essential oil	*Eugenia patrisii*	Identified compounds: sesquiterpene hydrocarbons (85.7%) E-caryophyllene (32.0%),bicyclogermacrene (10.0%), acyclic oxygenated sesquiterpenes DPPH: 146.2 mgTE/mL	AGP01 cell line	0.4 and 25 μg/mL for 72 h.	Cytotoxic effect IC50: 3.2 μg/mL and SI: 1.1	[41]

Composition results are represented in extract/oil dry weight. Meanings: ↑: increased, and ↓: decreased in comparison to the control group or sample. Abbreviations: Bax: Proteína X associada a bcl-2; Bcl-2: B-cell lymphoma 2; CDK4: Cyclin-dependent kinase 4; Cytc: Cytochrome c; c-Myc: Cellular Myc; DPPH: 2,2-diphenyl-1-picrylhydrazyl assay; HPLC: High-Performance Liquid Chromatography; IC_50_; concentration required for 50% inhibition; IL11: interleukin-11; JAK1: Janus kinase 1; JAK2: Janus Kinase 2; Pgui-1: Psidium guineense;Pgui-2: Psidium guineense; p-STAT3: Phospho-Stat3; STAT3: signal transducer and activator of transcription 3; SI: Selectivity Index; TPC: total phenolic compounds; XIAP: X-linked inhibitor of apoptosis protein.

### Açaí (*Euterpe oleracea*)

*Euterpe oleracea* Mart. is a palm tree that gives rise to açaí, a fruit found mainly in the Amazon region [19]. The fruit is rich in anthocyanins, which provides its purple color. In addition to anthocyanins, other phenolic compounds and flavonoids, such as ferulic acid, vanillic acid, catechin and quercetin, are also present in this species, making them known for their potent antioxidant, antimicrobial, anti-inflammatory, and anticancer capacity [19, 20]. The effect of açaí has been investigated in SC, by means of AGP01 cells. In this study, the cytotoxic and genotoxic impact of the clarified açaí pulp extract was evaluated. A juice was obtained from the fresh fruit, which was microfiltered and then centrifuged. The extraction method was not detailed in this study. The compounds orientin, homoorientin, taxifolin deoxyhexose, cyanidin 3-glycoside (C3G) and cyanidin 3rutinoside were identified in the extract [21]. After a short exposure to the extract, there was no reduction in cell viability. However, after a 72 h exposure, a 20.73% reduction was identified in the treated cells. Despite the antiproliferative effect, the extract did not induce apoptosis or necrosis in the cells. In addition, a significant reduction in the migratory capacity of the cells was seen at all concentrations tested, with no evidence of genotoxicity [21].

The literature describes those phenolic compounds, such as quercetin, resveratrol, curcumin, epigallocatechin gallate and anthocyanins, which are widely recognized for their antioxidant activity in normal cells, can paradoxically act as pro-oxidants in tumor cells [21, 22]. Through their pro-oxidant effect, they can induce oxidative stress and trigger cell death mechanisms, including autophagy [23]. Autophagy is a highly regulated process of degradation and recycling of damaged cellular components, often activated in response to adverse conditions [21, 23]. In tumor cells, the interaction between reactive oxygen species (ROS) and autophagy is complex and can either promote cell survival by eliminating ROS or induce cell death by uncontrolled oxidative stress [21, 23]. In the study carried out by Santos et al. (2024) [21], although no specific morphological analyses were carried out to detect autophagy in AGP01 cells, the absence of apoptosis and necrosis suggests that the reduction in viability observed in the cells may be associated with the activation of this mechanism [21]. However, further studies are needed to elucidate this hypothesis and determine the role of autophagy in the cellular response to the phenolic compounds investigated [21].

The anti-inflammatory and anti-tumorigenic effect of açaí was also investigated in C57BL/6 mice subjected to chronic colonization by *Helicobacter felis* in their stomachs, with the aim of evaluating its influence on modulating the inflammatory response and tumor development associated with the infection [24]. Freeze-dried açaí pulp was included in the diet of the mice, which were fed the experimental diet for periods of 4, 24 and 52 weeks. In contrast to the results in cells, the results of this study indicated that açaí had no inhibitory effect on gastric carcinogenesis caused by chronic infection with *Helicobacter felis*. The expression of the pro-inflammatory cytokines TNF-α and interleukin-1β showed no significant changes [24]. In other types of cancer, açaí has also shown chemopreventive effects [25]. Açaí pulp was incorporated into the diet of rats with oesophageal cancer, which received a diet containing 5% freeze-dried açaí pulp. The results showed a reduction in Interferon gamma (IFNγ) and interleukin 5 (IL-5) and the rat analog for human Interleukin-8 (GRO/KC). Human IL-8 is known to be a macrophage-derived mediator of angiogenesis [25]. The ability of all types of berries to reduce serum levels of IL-5 and GRO/KC may therefore be related to their inhibitory effect on esophageal tumorigenesis [25]. In colorectal cancer, açaí decreased myeloperoxidase (MPO) and cytokines such as tumor necrosis factor alpha (TNF-α), interleukin 1 beta (IL-1β) and interleukin 6 (IL-6), and inhibited cyclooxygenase 2 (COX-2), also reinforcing its anti-inflammatory effect. In addition to suppressing markers involved in cell survival, such as proliferating cell nuclear antigen (PCNA), açaí also favored apoptosis by increasing Bcl-2 Associated Agonist Of Cell Death (Bad) and cleaved caspase-3 and decreasing B-cell lymphoma 2 (Bcl-2) [26].

Cyanidin-3-O-glucoside (C3G), one of the main anthocyanins found in high concentrations in açaí, has been widely investigated for its anticancer effects in different tumor models, including breast cancer, colon cancer and glioblastoma [27]. Evidence suggests that its mechanisms of action involve inducing apoptosis, modulating the cell cycle and inhibiting angiogenesis, highlighting its therapeutic potential in controlling tumor progression [28]. The C3G was evaluated for its anticancer potential in various SC cell lines (MKN-45, MKN-28, MKN-74, KATO-3, AGS, HGC-27, NCI-N87, YCC-1, BGC-823, MGC-803, SGC-7901 and YCC-6). The cells were exposed to concentrations of 30, 60, 90, 120 and 150 μM of the compound for a period of 24 h, allowing analysis of its cytotoxic and regulatory effects on tumor progression [28]. The proliferation of all tested cells decreased. MKN-45 cells showed the greatest sensitivity to treatment, with an IC50 of 87 μM, and were therefore selected to investigate the effects of C3G on apoptosis, the cell cycle and cell migration [28]. Treatment with C3G promoted a significant increase in the apoptotic rate, from 0.17% to 56.26%, accompanied by morphological changes, such as cell rounding and fragmentation, evidenced by brightfield microscopy. In addition, the activation of pro-apoptotic proteins was observed, including Bad, cytochrome C, cleaved caspase-3 and cleaved PARP, while the expression of Bcl-2, an anti-apoptotic regulator, was reduced. These findings indicate that C3G positively modulates apoptosis in MKN-45 cells, reinforcing its therapeutic potential [28]. The cell cycle is a highly organized and controlled process, and is a crucial target for the development of anti-cancer therapies. Thus, some compounds have been widely explored in the treatment of cancer due to their ability to modulate and interrupt the progression of the cell cycle [28]. C3G decreased the number of cells in the G0/G1 phase in 19.64%, and increased the number of cells in the G2/M phase in 6.64%. In addition, a reduction in the expression levels of protein kinase B (AKT), cyclin-dependent kinase 1(CDK1), cyclin-dependent kinase 2 (CDK2) and cyclin B1 was observed, while the expression of p27 was significantly increased [28]. These findings suggest that C3G induces cell cycle arrest in the G2/M phase in MKN-45 cells, probably through modulation of the AKT signaling pathway, mediated by oxidative stress induced from ROS. Epithelial-mesenchymal transition (EMT) is a fundamental process for tumor progression, directly contributing to invasion and the formation of metastases. In this sense, C3G reduced the migration capacity of MKN-45 cells, treatment led to decreased expression of β-catenin, a key regulator of EMT, resulting in suppression of N-cadherin and vimentin, while E-cadherin levels were increased [28].

### Araçá (*Psidium spp*)

Araçá (*Psidium cattleianum* Sabine), also known as yellow araçá, red araçá, and pink araçá, is a little explored native Brazilian fruit that belongs to the Myrtaceae family [29]. The tree of this species is described as an evergreen shrub that produces oval fruits weighing up to 20 grams and containing multiple seeds. In addition to its sweet and characteristic flavor, the fruit stands out for its wide variety of compounds, such as anthocyanins, gallic acid, epicatechin, and ellagic acid, which confer high bioactivity and various benefits to human health [30]. Araçá leaves have been little studied, although they are rich in phenolic compounds such as gallic acid, quercetin, and protocatechuic acid [31]. Phenolic compounds and flavonoids, found in significant concentrations in araçá, are closely linked to its antioxidant capacity and protective effects against free radicals. Studies have shown that consuming araçá can slow down the progression of diseases such as diabetes, atherosclerosis, cardiovascular disease, and cancer [32].

The extract obtained from araçá leaves was evaluated in stomach cancer cells. In this study, the araçá leaves were air-dried and then ground. Extraction was carried out with 80% methanol at room temperature for three days under agitation [33]. The extract was filtered and the solvent was removed using a rotary evaporator. After complete removal of the solvent, the methanolic extract was lyophilized and suspended in water, with subsequent fractionation with n-hexane, chloroform, ethyl acetate and n-butanol. The compounds polymethoxylated flavone, sesquiterpenes, genistein, ferulic acid and oleanolic acid were identified in this extract [33].

The authors of this study, used SNU-16 cells to evaluate the antiproliferative effects of this extract. The results showed that the extract reduced the viability of the cells after three days of treatment, in a dose-dependent manner [33]. The reduction in the viability of SNU-16 cells is associated with the activation of the apoptosis process. In addition, the extract increased the population of cells in the sub-G1 phase [33]. Furthermore, the apoptosis mechanism is regulated by several proteins that are known to be anti-apoptotic, such as Bcl-2, extra-large B-cell lymphoma (Bcl-xL), Myeloid Leukemia 1 (MCL-1) and pro-apoptotic, BH3 Interacting Domain Death Agonist (Bid), BCL-2 associated protein X (Bax) and Caspases. Araçá leaves extract induced apoptosis by decreasing BCL-2 and increasing Caspase-3 and Caspase-8 protein levels, but did not alter Bax levels [33].

The antiproliferative and apoptotic capacity of the araçá extract can be explained by the presence of flavonoids such as polymethoxyflavone and genistein [33]. Genistein is a compound recognized for its antitumor and apoptosis-inducing effects. In this sense, primary gastric cancer cells were treated with different concentrations of genistein over a period of 24–72 h [34]. Cell death was evidenced according to the dose and exposure time. Apoptosis was perceived morphologically in the cells through chromatin condensation and nuclear fragmentation [34]. This was supported by the apoptotic index, which increased according to the time of exposure to genistein. In addition, modulation of the Bcl-2/Bax ratio was evidenced, since there was a reduction in Bcl-2 protein levels and an increase in Bax, which confers a favorable mechanism for the apoptotic process [34]. In SC cells with different characteristics (MGC-803 and SGC-7901), genistein also inhibited the ability of these cells to form colonies and self-renew. In addition, it reduced the size and weight of xenografted tumors in BALB/C nude mice when compared to those that did not receive the compound [35]. It also blocked the G2/M cell cycle by up-regulating tensin homologous phosphatase (PTEN), a tumor suppressor gene, in different SC cell lines (SGC-7901 and BGC-823) [36]. Gallic acid, another compound from the phenolic class, has also shown promising effects on SC. In AGS cells, gallic acid reduces the metastasis and invasive growth of gastric cancer cells by promoting the expression of RHOB ras homolog family member B (RhoB), negatively regulating the signaling of the AKT/GTPase pathway and inhibiting the activity of nuclear factor kappa B (NF-Κb) [37]. RhoB is a tumor suppressor protein with the ability to regulate factors associated with cell growth, migration and apoptosis. A decrease in RhoB levels is capable of enabling cell migration and invasion, as well as favoring tumor progression and metastasis [38]. In the study conducted by Ho et al. (2013) [37], gallic acid increased Rhob levels in AGS cells, which supports its promising effect in the positive regulation of this tumor suppressor gene [37].

*Psidium guineense Sw*, another native Brazilian species, is also known as araçá-do-campo [39]. The fruit is used in a variety of preparations and can be eaten fresh or used in desserts, drinks, ice cream and liqueurs [39]. The leaves of the araçá do campo have shown potent anti-inflammatory action, as well as storing volatile oils in their cavities. Thus, phenolic compounds and phytosterols have already been identified in the extract of the araçá do campo leaf. Monoterpenes and sesquiterpenes are predominant in the essential oil [39]. The essential oils obtained from the leaves of araçá-do-campo collected in different regions of the Amazon showed antiproliferative effects against AGP01 cells. The essential oil was obtained from leaves which were air-dried for two days at room temperature. The dried leaves were then crushed and subjected to hydrodistillation for three hours using a Clevenger. Finally, the oils were dried using anhydrous sodium sulphate to remove the moisture [40].

Monoterpenoids (limonene and α-pinene) and sesquiterpenes (E-caryophyllene) were the main compounds found in these essential oils [40]. Despite having been collected in different regions, the two samples showed similar chemical profiles in their composition. However, the sample with the highest concentration of E-caryophyllene showed the best cytotoxic effect against AGP01 cells. The Pgui-1 sample had an IC50 of 8.2 μg/mL, while the Pgui-2 sample had an IC50 of 15.7 μg/mL [40]. The lower the IC50 value, the more potent the compound or drug is in inhibiting cell viability [41]. E-caryophyllene, also known as β-caryophyllene, is a sesquiterpene widely found in various plants [42]. It is one of the main components of essential oils found in plants such as basil, oregano, cannabis and rosemary. This compound is approved as a flavoring by both the Food and Drug Administration (FDA) and the European Food Safety Authority (EFSA) [42]. Thus, its anticancer effects can be associated with various mechanisms, including the induction of apoptosis, the inhibition of angiogenesis and the suppression of tumor metastasis [42]. β-caryophyllene reduced cell proliferation in human B lymphocyte lines transformed by the Epstein-Barr virus (MoFir) and mouse lymphoma (BS-24-1), as well as activating caspase-3, a key protein in the apoptosis process. It also promoted the activation of endonucleases and the selective fragmentation of genomic DNA, acting specifically against tumor cells without affecting normal cells [43]. In agreement with these results, β-caryophyllene oxide also showed promising effects on SC cells of the SNU-1 and SNU-16 lineages. The inhibition of cell proliferation occurred in a dose-dependent manner, with IC50 of 16.79 μM and 27.39 μM, respectively [44]. The presence of these compounds in araçá leaves reinforces its considerable therapeutic potential, highlighted by its anticancer properties. This bioactive profile positions the araçá species as a promising natural source for research and development of new therapeutic agents [45].

### Cocoa (*Theobroma cacao*)

The cocoa tree (*Theobroma cacao*), which belongs to the Malvaceae family, is a tree that produces the fruit known as cocoa. The seeds, also known as cocoa beans, are the main ingredients for chocolate production [46]. In addition to their unique and attractive flavor, cocoa beans contain a variety of bioactive compounds such as polyphenols, flavonoids, alkaloids, phytosterols, and fatty acids. The presence of these compounds is directly related to beneficial effects on human health, due to their antioxidant, anti-inflammatory, and anticancer properties [46]. According to the International Cocoa Organization (ICCO), more than 4,000 tons of cocoa beans are processed and roasted worldwide every year [47]. This process results in the generation of a considerable amount of cocoa bean husks, which are largely discarded as waste by this industry. However, this underutilized fraction is rich in bioactive compounds such as catechin, epicatechin and procyanidin [47].

To date, only one study has been carried out exploring the effects of cocoa on SC. In an *in vitro* model of SC, a potential antiproliferative effect of cocoa bean extracts on SNU1 cells was observed [48]. The extracts were produced from 50 g of the cocoa bean shell and 50 g of the cocoa bean. The samples were extracted by means of different solvents: methanol, ethanol and acetone 50% (v/v), under reflux for five hours at 60°C. The extracts were filtered through filter paper, evaporated to remove the solvent and freeze-dried [48]. The extract obtained using an aqueous solution of 50% (v/v) acetone showed a higher concentration of polyphenols (33.5%) and greater efficacy in inhibiting cell proliferation, which suggests that the antiproliferative effect is associated with the presence of polyphenols [48]. In the same study, cocoa bean extract inhibited the suppression of gap junction intercellular communication (GJIC) in WB-F344 cells [48]. GJICs are crucial biochemical markers in carcinogenesis, especially during the cancer-promoting phase. Thus, substances capable of blocking GJIC have the potential to interrupt this phase of carcinogenesis, thus contributing to the prevention, delay, and inhibition of tumor development. The anticancer effect of the compounds found in cocoa beans is also associated with their ability to prevent oxidative damage in cells by eliminating free radicals, preserving antioxidant proteins, preventing the formation of nitrosamines, and/or inducing apoptosis by blocking the phosphoinositide 3-kinase (PI3K)-Akt signaling pathway [49–51]. However, more studies are needed to elucidate the mechanisms involved in the chemopreventive action of cocoa in SC.

### Coriander (*Eryngium foetidum*)

Coriander (*Eryngium foetidum*), also known as caboclo chicory or Brazilian coriander, is a member of the Apiaceae family, a leafy plant widely cultivated in Brazil, especially in the North, where it plays an important role as a spice in local cuisine [52, 53]. Classified as an unconventional food plant, this Amazonian species is notable for its high content of bioactive compounds, including chlorogenic acid, ferulic acid, lutein and β-carotene, highlighting its nutritional and functional potential [52]. Thus, the anticancer effect of this plant was investigated in SC cell lines with different morphologies (SGC-7901, MGC-803 and AGS) and in a non-tumor cell line (GES-1) [54].

The plants were air-dried and cut into small pieces, and then liquid nitrogen was added for grinding and sieving. The aqueous extract was produced from dried plants, which were boiled with continuous stirring for 30 min and cooled to room temperature, then centrifuged, filtered and freeze-dried and stored at −20°C. The powdered leaves were also extracted using ethanol, n-hexane and petroleum ether and then filtered. The solvent was removed by rotary evaporation [54]. Cell viability was assessed using extracts obtained with different solvents (ethanol, n-hexane, petroleum ether and water). After exposure for 72 h, the ethanolic extract had the best effect on reducing cell viability, particularly in SGC-7901 cells, with no change in GES-1 cells [54]. In addition, it induced the interruption of the cell cycle of cells in the G0/G1 phase, mediated by the negative regulation of the cyclin D1 and cyclin-dependent kinase 4 (CDK4) proteins [54].

Apoptosis has a fundamental role to play in the elimination of tumor cells, by inhibiting proliferation, inducing changes in cell morphology and promoting DNA fragmentation. Coriander altered the morphology of SGC-7901 cells, where typical apoptotic features were predominant in the group exposed to the extract [54]. The cells showed nuclear shrinkage, condensation and chromatin fragmentation [54]. In addition, the percentage of apoptotic cells also increased in the treated group when compared to the control. A decrease in mitochondrial membrane potential (MMP) was detected in the cells in a dose-dependent manner, indicating its close relationship with the apoptotic process and corroborating the pro-apoptotic effects of coriander [54]. In addition, increased levels of cytochrome C (Cyt c) were found, which is a molecule released from the mitochondria into the cytoplasm in response to the loss of mitochondrial membrane potential. Negative regulation of Bcl-2 was seen, while Bax and caspase-3 showed significant activation. These results indicated that apoptosis was activated via the mitochondrial pathway [54].

### Guava (*Psidium guajava*)

Guava (*Psidium guajava L*.) is a fruit native to Brazil, belonging to the Myrtaceae family, which is also commonly found in other tropical regions. The fruit has a variety of nutrients and bioactive compounds, such as fiber, vitamins, minerals, polyphenols, flavonoids, terpenoids, essential oils and unsaturated fatty acids [55]. Guava leaves are used to treat diseases such as diabetes, wounds, ulcers and rheumatic pain [56]. The biological effects are linked to the phenolic compounds such as, flavonoids, tannins, triterpenoids and sesquiterpenoids that are present in the leaves, which give it antioxidant, anti-inflammatory and anticancer action [55].

Guava leaves were used to produce an essential oil which was investigated in AGP01 cells. The essential oil was produced from leaves, which were air-dried for two days at room temperature. The leaves were then crushed and subjected to hydrodistillation for three hours using a Clevenger. The oils were dried using anhydrous sodium sulphate to remove the moisture [40]. The essential oil extracted from guava leaves showed antiproliferative activity in AGP01 cells, with an IC50 of 16.3 μg/mL. In addition, this essential oil exhibited potent antioxidant activity (195.7 mgTE/mL) attributed to the high concentration of sesquiterpenes, with β-caryophyllene standing out as one of the main compounds present [40].

β-Caryophyllene and its derivative, β-Caryophyllene oxide, both demonstrate significant cytotoxic activity in different types of cancer [57]. β-caryophyllene demonstrated significant inhibition of cell proliferation in two colon cancer cell lines, HT-29 and HCT-116, as well as in the pancreatic cancer cell line PANC-1 [57]. However, in CaCo-2 intestinal cancer cells, β-caryophyllene did not exhibit a significant impact on decreasing cell proliferation. Additionally, a study in C57BL/6N mice, previously inoculated with melanoma cells, revealed that β-caryophyllene attenuates the pro-carcinogenic effects of a high-fat diet, reinforcing its potential in modulating the tumor microenvironment. The mechanism of action of this compound is related to increased production of ROS and loss of mitochondrial membrane potential. This process occurs due to increased expression of Bax and reduced expression of Bcl-2 [57]. The oligomerization of Bax in the outer mitochondrial membrane leads to the formation of pores, increasing its permeability and promoting the release of Cyt c into the cytoplasm, a key event in the activation of the intrinsic apoptosis pathway [57, 58]. The presence of Cyt c in the cytosol leads to the formation of the apoptosome, which activates caspase-9 and triggers the cascade of effector caspases, culminating in programmed cell death [57, 58].

β-Caryophyllene oxide interacts with nucleophilic groups in DNA, hindering its replication and, consequently, promoting the death of cancer cells with a high proliferation rate. Thus, its binding to DNA represents one of the mechanisms that may be related to its cytotoxic activity [59]. β-Caryophyllene oxide demonstrated dose-dependent inhibition of the proliferation of PC-3 prostate cancer cells and MCF-7 breast cancer cells [42]. In addition to inducing the generation of ROS, it activated the mitogen-activated protein kinase (MAPK) pathway and suppressed PI3K/AKT/mTOR/S6K1 signaling, an essential axis for cell survival, proliferation and tumor angiogenesis. Additionally, it significantly reduced the levels of key proteins in cancer progression, including those involved in cell proliferation (cyclin D1), metastasis, cyclooxygenase-2 (COX-2), angiogenesis, vascular endothelial growth factor (VEGF), and resistance to apoptosis, such as Bcl-2, Bcl-xL, IAP-1, IAP-2 and survivin [42].

### Jambu (*Acmella oleracea*)

Jambu (*Acmella oleracea*), also known as watercress from Pará, is a species native to the Amazon region [60]. Jambu leaves have been used in typical regional dishes, such as tacacá. In addition to its traditional use in cooking, jambu flowers and leaves are also used in traditional medicine [60]. The biological effects of jambu are associated with its anti-inflammatory and antioxidant activity, related to the species’ main compound, spilanthol. The effect of jambu has been investigated in breast cancer and cervical cancer, but there are still few studies on SC [60].

A study using AGP01 cells investigated the effects of the hydroalcoholic extract of jambu and spilanthol [61]. The jambu hydroalcoholic extractwas obtained from flowers which were extracted using a 7:3 ethanol/water solution using a soxhlet, with the aid of a heating mantle and left to reflux for 4 hours. The extract was concentrated and the solvent was removed using a rotary evaporator at 60°C. To isolate spilanthol, the extract was fractionated using a C18 SPE cartridge with methanol as the eluent, generating four fractions. Fraction 2 was purified using preparative HPLC with a C18 column, under gradient elution of water and acetonitrile [61].

The extract and the isolated compound inhibited cell proliferation, especially spilanthol after 24 h of treatment [61]. To investigate the possible mechanistic pathway involved, the authors carried out *in silico* analysis of the JAK1 and JAK2 proteins (Janus Kinases) of the JAK/STAT pathway in SC. Spilanthol interacted with the proteins forming strong bonds, which may inhibit the phosphorylation of JAK proteins and activation of signal transducer and activator of transcription 3 (STAT3) [61].

The JAK family of proteins plays a crucial role in cell survival, proliferation, differentiation and apoptosis. Yet its activation, as well as associated mutations, is frequently observed in gastric cancer [62]. Similarly, the STAT family is known to be involved in cell differentiation, cell cycle control and the regulation of cell death. It has also been reported that the STAT family contributes to tumor progression, as well as being associated with resistance to certain chemotherapy drugs [62]. In particular, chronic activation of STAT3 is a key factor in the development of SC. When activated, STAT3 stimulates the production of pro-inflammatory cytokines, such as tumor necrosis factor (TNF), IL-1β, IL-6 and IL-22, which play a central role in regulating inflammation and may favor tumorigenesis. For this reason, STAT3 is often found in its active form in gastric cancer cell lines. However, its inhibition can promote apoptosis, representing a therapeutic strategy [62].

### Physalis (*Physalis angulata* linn)

*Physalis angulata*, popularly known as physalis, is an edible fruit from the Solanaceae family, widely distributed in the Amazon region [63]. Characterized by its sweet and pleasant taste, physalis, can be eaten both fresh and in processed products, standing out for its gastronomic and functional potential [63]. The secondary metabolites present in this fruit play a fundamental role in its use in traditional medicine. These bioactive compounds are associated with the fruit’s therapeutic properties and have been widely studied for their potential anti-inflammatory, antioxidant, anti-parasitic and anti-cancer effects [64].

**Figure 3:**
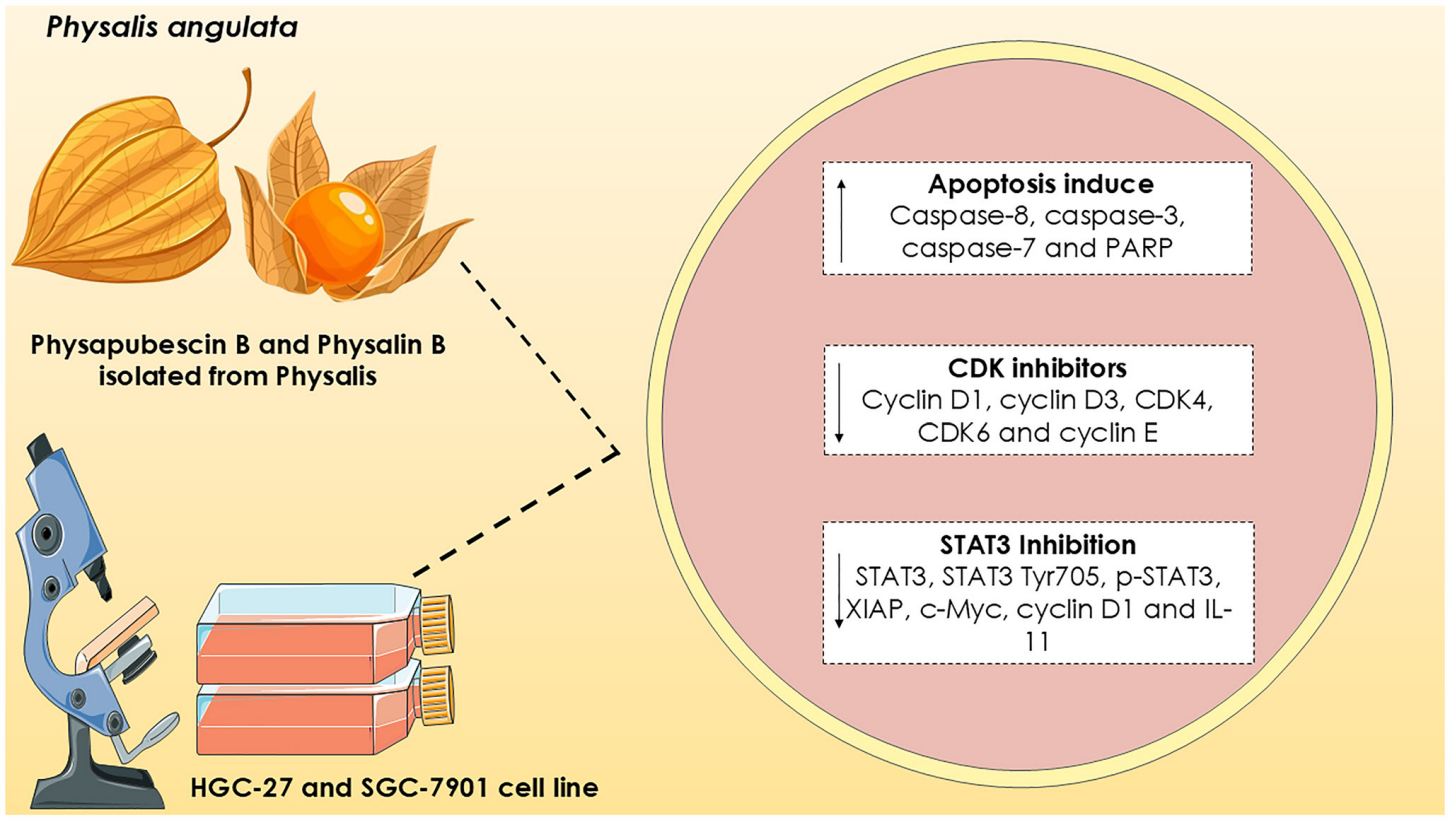
Effects of compounds isolated from Physalis on HGC-27 and SGC-7901 stomach cancer cell lines. According to Fang et al. (2022), Physalin B suppressed the expression of cell cycle regulatory proteins, including cyclin D1, cyclin D3, CDK4, CDK6 and cyclin E, as well as increasing the protein levels of caspase-8, caspase-3, caspase-7 and PARP, promoting apoptosis. According to Dai et al. (2020), Physapubescin B inhibited cell proliferation and induced apoptosis in gastric cancer cells by suppressing the phosphorylation of STAT3 and the expression of downstream targets such as STAT3, STAT3 Tyr705, p-STAT3, XIAP, c-Myc, cyclin D1 and IL-11. Meanings: ↑: increased, and ↓: decreased. Abbreviations: CDK4: Cyclin-dependent kinase 4; CDK6: Cyclin-dependent kinase 6; c-Myc: Cellular Myc; IL11: interleukin-11; PARP: poly(ADP-ribose) polymerase); p-STAT3: Phospho-Stat3; STAT3: signal transducer and activator of transcription; XIAP: X-linked inhibitor of apoptosis protein. Own authorship using open-access illustrations under CC BY 4.0 license from smart.servier.com and canva pro.

Physalin B is a physalis-derived compound belonging to the secosteroid class that has already shown promising effects in lung, breast, prostate and melanoma cancer [65]. To investigate the effect of fisalin B on breast cancer, two cell lines (SGC-7901 and HGC-27) were used, however, the method for extracting and isolating the compound was not detailed in this study [65]. After prolonged exposure of 72 h, physalin B reduced cell proliferation with IC50 of 9 μM, in addition to a decrease in colony formation of these cells. Additionally, a dose-dependent reduction in cell cycle regulatory proteins was found, including cyclin D1, cyclin D3, CDK4, cyclin-dependent kinase 6 (CDK6) and cyclin E (Figure 3), suggesting a potential inhibitory effect on cell cycle progression [65]. Caspase-dependent activation of apoptosis was also observed, with a significant increase in the cleavage of caspase-8, caspase-3, caspase-7 and poly (ADP-ribose) polymerase (PARP) (Figure 3). These results showed that physalin B induced programmed cell death in a caspase-dependent manner, further strengthening its potential as a promising therapeutic strategy in the treatment of gastric cancer [65].

In addition, physapubescin B, another compound isolated from Physalis angulata, showed promising activities in the treatment of SC. Physapubescin B was able to induce apoptosis in the MGC803, HGC27 and MKN45 cell lines, as well as negatively regulating STAT3 expression [66]. STAT3 activation is associated with increased interleukin levels, with IL-11 being a crucial cytokine for STAT3 activation and tumor progression (Figure 3) [66]. However, IL-11 levels were significantly reduced in the cells after physapubescin B treatment. In an *in vivo* xenograft model, physapubescin B resulted in a 31.4% reduction in tumor volume in mice treated with 40 mg/kg via intravenous injection. These results reinforced the therapeutic potential of physapubescin B in SC [66]. Withangulatin I, a steroid isolated from Physalis angulata, was investigated at SC. This compound was extracted and isolated from dried and ground whole plant using methanol at room temperature for one week [67]. The concentrated extract was fractionated by silica gel column chromatography, using a mixture of chloroform/acetone in increasing polarity as the mobile phase, with the fractions being monitored by thin layer chromatography (TLC). The fractions, containing witanolides, were then subjected to final purification by preparative TLC, revealed with the mobile phase n-hexane/ethyl acetate/methanol, allowing the isolation of the compound Withangulatin I [67]. Thus, Withangulatin I, demonstrated potent ability to inhibit the proliferation of AGS cells, with an IC50 value = 1.8 μM. However, this study did not investigate the mechanistic pathway involved in the antitumor action of withangulatin I [67].

### Pitanga (*Eugenia uniflora*)

Pitanga (*Eugenia uniflora*), known as Brazilian cherry, is a fruit produced by the pitangueira, a tree native to Brazil that also belongs to the Myrtaceae family. Its fruit has a sweet-acidic flavor and can be consumed fresh or as a source of ingredients for other food products such as jams, jellies, liqueurs and juices [68]. Its composition is rich in polyphenols, vitamins and minerals, which gives this species great bioactivity [69]. In addition to the fruit, the leaves of the pitangueira have natural compounds that give it interesting biological properties [70]. The anticancer effect of essential oils from five pitanga species from the Amazon region was evaluated on SC cells (AGP-01). The essential oils were extracted by hydrodistillation, followed by drying with anhydrous sodium sulphate to remove residual moisture [71]. In this study, only two samples at concentrations of 0.4–25 μg/mL showed antiproliferative effects against the tested lineage, with IC50 of 12.60 μg/mL and 8.73 μg/mL. These are the samples with the highest concentration of oxygenated sesquiterpenes and sesquiterpene hydrocarbons, and which also showed the best antioxidant capacity, which justifies their anticancer action [71].

Other researchers have also investigated pitanga leave extract. In this study, the pitanga leaves were air-dried for thirteen days [72]. Subsequently, the leaves were ground and macerated in ethanol for ten days, and the extract was filtered and concentrated in a rotary evaporator. The ethanolic extract was suspended in distilled water and partitioned with dichloromethane and ethyl acetate. After fractionation, the extracts were chemically characterized and the ethyl acetate and aqueous fractions were selected for biological tests [72]. Pitanga leaves extracts were therefore evaluated for their activity on AGS cells and their potential anti*-Helicobacter pylori* effect, a bacterium associated with the development of SC. Three extracts obtained by extraction with ethanol, ethyl acetate and water were investigated [72]. Among them, only the aqueous extract showed the ability to reduce the cell viability of AGS cells. However, the ethyl acetate extract and the aqueous extract were able to inhibit the growth of *H. pylori*. Phytochemical analysis revealed the predominant presence of ellagitannins in this extract [72]. Ellagitannins, known as water-soluble tannins, are bioactive compounds found in various plant matrices, such as blackberry, strawberry, pitanga and jabuticaba, and are mainly concentrated in the seeds of these fruits [72, 73]. These compounds act directly on the elimination of nitric oxide, the elevation of which can trigger inflammatory processes and oxidative stress, crucial factors in the development of cancer. In addition, ellagitannins have shown the potential to reduce and inhibit the proliferation of cancer cells, acting through different molecular mechanisms, including the activation of apoptosis through intrinsic and extrinsic pathways [73].

### Ubaia (*Eugenia patrisii*)

Ubaia (*Eugenia patrisii*) is a fruit native to the Amazon that belongs to the Myrtaceae family, also known as “fruta do mato” or “ubaia rubi”. It is not yet widely marketed or known, but can be eaten fresh or in the form of juices, jams and jellies [74]. Ubaia leaves are used to produce essential oil. Also, previous studies have reported a composition rich in hydrocarbon sesquiterpenes and oxygenated sesquiterpenes, with Germacrene, Bicyclogermacrene, and E-caryophyllene being the main compounds found in its leaves [74]. Ubaia essential oil was investigated for its cytotoxic potential in SC cells. The leaves of the plant were air-dried for two days at room temperature, crushed and subjected to hydrodistillation for three hours using a Clevenger-type apparatus. The oil was then dried with anhydrous sodium sulphate to remove residual humidity. The essential oil showed antiproliferative effects on AGP01 cells, with an IC50 value of 3.2 μg/mL. The main compound found in ubaia essential oil, E-caryophyllene, has already shown cytotoxic effects against other cancer cell lines, such as melanoma in previous studies (SKMEL-19) and colon (HCT116) [40].

## KNOWLEDGE GAPS AND FUTURE PERSPECTIVES

Several factors, such as the method of preparation, botanical origin and cultivation conditions, can directly influence the chemical composition of extracts and, consequently, their biological effects [75]. This aspect becomes even more critical in complex products such as plant extracts, in which multiple compounds can act synergistically. Thus, small variations in the profile of primary and secondary metabolites can result in different biological responses. In view of this, we highlight the importance of detailed chemical characterization of these extracts, using robust analytical techniques and integration with consistent biological data [75]. In this context, more modern approaches that combine chemical analysis, standardized bioassays and computational tools have proven effective for identifying bioactive compounds and their possible interactions, even without the need for complete isolation [75]. We therefore stress the importance of incorporating these strategies into future investigations in order to increase the reliability and applicability of the findings. Although Brazilian plant species show promising potential as therapeutic agents in SC, the available studies are still scarce.

Most of the studies presented in this review were carried out on cell models and they did not detail the molecular pathways and underlying mechanisms, which represents an important limitation. Only four investigations detailed the underlying pathways of action. Among these studies, we highlight the analysis of araçá, coriander and physalin B on apoptotic markers, as well as jambu in the modulation of inflammatory markers associated with SC. Despite these limitations, the existing data, although still initial, suggests a relevant chemopreventive potential. Thus, these findings can provide as a starting point to encourage further scientific exploration of plant species from Brazil’s vast biodiversity. By gathering and critically analyzing this information, this review seeks not only to consolidate current knowledge, but also to stimulate new research that values Brazil’s biological heritage and contributes to the development of innovative strategies for the prevention and treatment of SC as an adjunct therapy.

## CONCLUSIONS

Brazil has a rich variety of plant species, making part of an immense biodiversity, and also many of which are still little explored, especially in the SC. Although, there are studies of these species, they are still limited, the available data indicate excellent therapeutic potential, minimizing or slowing down the progression of SC, and suggesting an action as an adjunct therapy. Among the plant parts analyzed, the less investigated fractions, such as leaves and peel, have shown promising effects. Most of the research don’t focus on molecular pathways involved in progression, cell proliferation, and apoptosis in cellular models of SC, what certainly is a limitation in this review. The large amount of studies utilized cell culture and also there is only two pre-clinical study. However, in order to elucidate the underlying mechanisms and accurately assess the efficacy of these species in SC therapies and/or prevention, more research is needed, both *in vitro* and *in vivo*. The valorization and use of these underestimated species not only could contribute to the recognition of Brazilian flora, but also to advance the Sustainable Development Goals, promoting public health in a sustainable and innovative way.

## Abbreviations

AKT: protein kinase B
Bad: BCL-2 Associated Agonist Of Cell Death
Bax: BCL-2 associated protein X
Bcl-xL: extra-large B cell lymphoma
BID: BH3 Interacting Domain Death Agonist
SC: Stomach cancer
CDK2: Cyclin-dependent kinase 2
CDK4: Cyclin-dependent kinase 4
CDK6: Cyclin-dependent kinase 6
Cyt c: Cytochrome C
COX-2: Cyclooxygenase 2
C3G: cyanidin 3-glucoside
DNA: Deoxyribonucleic acid
EFSA: European Food Safety Authority
EMT: Epithelial-mesenchymal transition
FDA: Food and Drug Administration
GJIC: Gap junctional intercellular communications
IC_50_: Concentration required for 50% inhibition
ICCO: International Cocoa Organization
IFNγ: Interferon gamma
IL-5: Interleukin 5
IL-1β: interleukin 1 beta
IL-6: Interleukin 6
IL-8: Interleukin 8
JAK: Janus Kinase
JAK1: Janus Kinase1
JAK2: Janus Kinase2
GRO/KC: Mouse analogue for human Interleukin-8
MoFir: Epstein-Barr virus-transformed human B lymphocytes
MPO: Mieloperoxidase
NF-Κb: Nuclear factor kappa B
PARP: Poly(ADP-ribose) polymerase
PCNA: Proliferating cell nuclear antigen
PI3K: phosphatidylinositol 3-kinase
PTEN: tensin homologous phosphatase
RhoB: RHOB ras homolog family member B
STAT: Signal transducer and activator of transcription
STAT3: signal transducer and activator of transcription 3
TNF: Tumor necrosis factor
TNF-α: Tumor necrosis factor alpha
VEGF: vascular endothelial growth factor
